# How to Apply Root Dressings

**Published:** 1904-06

**Authors:** W. R. Clark


### HOW TO APPLY ROOT DRESSINGS.

By W. R. Clark.

Dr. Lewis, of Austin, Minn., was in my office the other day and noticed me trying to pick the cotton off the end of a barbed broach by which I had been carrying some oil of cloves into a root. He said: "Let me show you a thing that Dr. Searl of Owatonna put me on to." He took a smooth broach, held it a moment in the flame, touched it to a piece of beeswax, and wrapped the cotton around it. The idea is a good one. You can carry the cotton to the end of the root, give a slight rotation of the broach, and leave it there, or, holding on to the shred, you can remove it with the broach, and remove it instantly. Try it.—*Cosmos.*

